# One-step of tryptophan attenuator inactivation and promoter swapping to improve the production of L-tryptophan in *Escherichia coli*

**DOI:** 10.1186/1475-2859-11-30

**Published:** 2012-03-02

**Authors:** Pengfei Gu, Fan Yang, Junhua Kang, Qian Wang, Qingsheng Qi

**Affiliations:** 1 State Key Laboratory of Microbial Technology, National Glycoengineering Research Center, Shandong University, Jinan 250100, People's Republic of China

## Abstract

**Background:**

L-tryptophan is an aromatic amino acid widely used in the food, chemical and pharmaceutical industries. In *Escherichia coli*, L-tryptophan is synthesized from phosphoenolpyruvate and erythrose 4-phosphate by enzymes in the shikimate pathway and L-tryptophan branch pathway, while L-serine and phosphoribosylpyrophosphate are also involved in L-tryptophan synthesis. In order to construct a microbial strain for efficient L-tryptophan production from glucose, we developed a one step tryptophan attenuator inactivation and promoter swapping strategy for metabolic flux optimization after a base strain was obtained by overexpressing the *tktA*, mutated *trpE *and *aroG *genes and inactivating a series of competitive steps.

**Results:**

The engineered *E. coli *GPT1002 with tryptophan attenuator inactivation and tryptophan operon promoter substitution exhibited 1.67 ~ 9.29 times higher transcription of tryptophan operon genes than the control GPT1001. In addition, this strain accumulated 1.70 g l^-1 ^L-tryptophan after 36 h batch cultivation in 300-mL shake flask. Bioreactor fermentation experiments showed that GPT1002 could produce 10.15 g l^-1 ^L-tryptophan in 48 h.

**Conclusions:**

The one step inactivating and promoter swapping is an efficient method for metabolic engineering. This method can also be applied in other bacteria.

## Background

L-tryptophan is an essential aromatic amino acid for humans and animals which can be used as food additive, infusion liquids, pellagra treatment, sleep induction and nutritional therapy [1,2]. Since the chemical synthesis of L-tryptophan has many disadvantages such as nonrenewable toxic raw materials and racemic mixtures of products, microbial fermentation of L-tryptophan has become attractive alternative. *E. coli*, a widely used production host that possesses clear genetic background, convenient metabolic engineering tools and fast growth in cheap media, has attracted many attentions for the production of L-tryptophan and other aromatic compounds [3-7].

**Figure 1:**
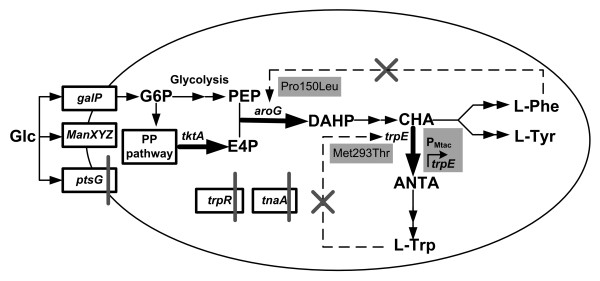
**The strategies for constructing the L-tryptophan producing strain GPT1002**. The shaded boxes represent genetic modification, and the gray bars indicate the genes that were deleted. Dotted lines indicate feedback inhibition. The black X indicates that the inhibition is removed. The thick black arrows indicate the increased flux or activity by directly overexpressing the corresponding genes in plasmids. Glc glucose, G6P glucose-6-phosphate, E4P erythrose-4-phosphate, PEP phosphoenolpyruvate, DAHP 3-deoxy-D-arabino-heptulosonate, CHA chorismate, ANTA anthranilate, L-Phe L-phenylalanine, L-Tyr L-tyrosine, L-Trp L-tryptophan, *tktA *transketolase, *aroG *3-deoxy-D-arabino-heptulosonate-7-phosphate synthase (phenylalanine repressible), *trpE *component I of anthranilate synthase, *trpR *trp operon repressor, tnaA tryptophanase, *ptsG *glucose-specific PTS enzyme IIBC components, *manXYZ *mannose-specific PTS enzyme IIABCD components, *galP *D-galactose transporter, PP pathway pentose phosphate pathway.

The biosynthesis of the L-tryptophan in *E. coli *begins with the condensation of phosphoenolpyruvate (PEP) and erythrose 4-phosphate (E4P) to form 3-deoxy-D-arabino-heptulosonate-7-phosphate (DAHP), and then proceeds to chorismate, a key intermediate product leading to the formation of L-tryptophan, L-tyrosine, and L-phenylalanine (Figure 1). In the L-tryptophan branch pathway, L-serine and phosphoribosylpyrophosphate (PRPP) are needed as well. Since the biosynthesis of L-tryptophan from glucose involves a long metabolic pathway, there are several regulatory circuits which influence the accumulation of L-tryptophan such as transcriptional repression, attenuation, feedback inhibition and so on [1,8]. Among these regulatory circuits, tryptophan attenuator is critical due to its sensitivity to the *in vivo *L-tryptophan level [9]. Therefore, removing or inactivating the tryptophan attenuator was supposed to be an effective method for elevating the L-tryptophan accumulation. Herry et al. identified a mutation in the tryptophan attenuator sequence from a hyperproducing strain of *Corynebacterium glutamicum *and proved its contribution to the deregulation of the tryptophan operon [10]. However, little attention had been focused on tryptophan attenuator to improve L-tryptophan production in *E. coli*.

Otherwise, the transcription and expression of tryptophan operon is pivotal to obtain high L-tryptophan accumulation as well [6]. Promoter swapping allowed researchers to replace a wild type promoter with the one that has been designed for a increased or controlled transcription strength while retaining the natural genetic context of a gene or an operon in the genome [11]. Consequently, by promoter swapping and engineering, the targeted metabolites can be elevated. For example, to maximize the threonine production, Lee at al. created an L-threonine producing strain by replaced three different chromosomal promoters. After replaced the native promoter of the *ppc *gene with *trc *promoter in the chromosome, the engineered strain showed a higher PPC flux than the wild type, and therefore resulting 27.7% increased threonine production [12]. In another study, Alper et al. found a correlation between promoter strength and lycopene production. By introducing a promoter library that was created by error-prone PCR into *E. coli *to replace native promoter of phosphoenolpyruvate carboxylase or deoxy-xylulose-phosphate synthase, they identified a suitable promoter for lycopene production [13].

In this study, we first constructed a basic L-tryptophan-synthetic strain by inactivation of the *trpR*, *tnaA *and *ptsG*, expressing in plasmids the feedback resistant *aroG*, *trpE *(*aroG^FR ^*and *trpE^FR ^*respectively), and *tktA *genes in wild *E. coli *K-12 W3110. Then, we inactivated the tryptophan attenuator and replacing the original *trp *promoter of tryptophan operon with a novel promoter cluster consisted of five core-*tac*-promoters aligned in tandem (5CP*tacs *promoter cluster) in one step. The resulting strain GPT1002 showed higher transcription of tryptophan operon genes and more L-tryptophan accumulation than the parent strain.

## Results and discussion

### Construction of the basic L-tryptophan-synthetic *E. Coli *GPT1001

The overall strategies for constructing L-tryptophan production strain are shown in Figure 1. To generate an *E. coli *that overproduces and excretes L-tryptophan, the following manipulation was done: First, *trpR *gene, which encodes a tryptophan transcriptional repressor, was knocked out to eliminate transcription regulation of the genes in L-tryptophan pathway [6,14]. Knockout of this gene slightly improved the tryptophan accumulation (Table 1). Second, *trpE *and *aroG*, encoding component I of anthranilate synthase and DAHP synthase, respectively, were cloned into the low-copy-number vector pCL1920 and were expressed in the *E. coli *(∆*trpR*). Since the expression of wild type *trpE *and *aroG *are feedback inhibited by L-tryptophan and L-phenylalanine, respectively, site-directed mutations of *trpE *(Met293Thr) and *aroG *(Pro150Leu) were performed in our study to remove the feedback inhibition [15,16]. The resulting recombinant *E. coli *(∆*trpR*) harboring the overexpressed and mutated *trpE *and *aroG *can produce 0.74 g l^-1 ^L-tryptophan in batch cultivation, which is 6000 fold higher than the wild type *E. coli *(Table 1).

**Table 1:** Development of L-tryptophan producing *E. coli *strains

Strain	L-tryptophan (mg l^-1^)
W3110	0.12 ± 0.01
W3110 (∆*trpR*::FRT)	0.14 ± 0.02
W3110 (∆*trpR*::FRT)/pCL1920-*trpE^FR^*	64.46 ± 2.17
W3110 (∆*trpR*::FRT)/pCL1920-*trpE^FR^*-*aroG^FR^*	736.83 ± 3.98
W3110 (∆*trpR*::FRT)/pTAT	1018.98 ± 1.89
W3110 (∆*trpR*::FRT, ∆*tnaA*::FRT)/pTAT	1188.20 ± 2.56
W3110 (∆*trpR*::FRT, ∆*tnaA*::FRT, ∆*ptsG*::FRT)/pTAT^a^	1208.82 ± 1.33

L-tryptophan titer in mg l^-1 ^reported was the final production obtained when glucose had been completely consumed in 50 mL fermentative medium containing 20 g l^-1 ^glucose, shaken at 250 rpm and 37°C.

^a ^*E.coli *GPT1001

Alleviating the feedback repression of the product increased the expression of the key enzymes in the tryptophan biosynthesis pathway, while provision of more precursors would enable the enhanced metabolic flux. *tktA *gene, encoding a transketolase in pentose phosphate pathway, and overexpression of this gene in *E. coli *was proved to supply more E4P, a precursor of L-tryptophan [17]. Otherwise, carbon flux distribution analysis at the node in wild *E. coli *indicated that phosphoenolpyruvate:carbohydrate phosphotransferase system (PTS) is the largest consumer of PEP, while the relative carbon flux directed to aromatic amino acid biosynthesis is only around 1.5% of the PTS consumed [18]. Therefore we knocked out *ptsG*, which encodes the IIBC component of glucose-specific PTS system, to provide more PEP. In our base strain, we performed modification of the host to increase the levels of precursors PEP and E4P, while PRPP and Lserine are also building blocks for L-tryptophan. Therefore, increasing the availability of L-serine by amplification of the deregulation *serA *gene [19] and PRPP by overexpresssion of *prs *and *ywlF *genes involved in the biosynthetic pathway of PRPP from ribulose-5-phosphate [20] should be useful for high L-tryptophan accumulation. Finally, we knocked out the gene *tnaA*, which encodes a tryptophanase that catalyzes the reaction of L-tryptophan back into indole [3]. The resulting L-tryptophan-synthetic strain was named GPT100. Then we transformed plasmid pTAT into *E. coli *GPT100 and constructed strain GPT1001. This strain was able to produce 1.3 g l^-1 ^L-tryptophan in batch cultivation and was therefore used as base strain for further experiment.

### One-step L-tryptophan attenuator inactivation and promoter swapping

The expression of tryptophan biosynthesis operon was negatively regulated by the attenuator downstream of the promoter operator site until tryptophan starvation is severe. However, simply removal of the attenuator probably cannot reach a sufficient expression of the tryptophan operon genes [21]. Therefore it is essential to improve the expression of genes in tryptophan operon at the same time of inactivating the attenuator.

**Figure 2:**
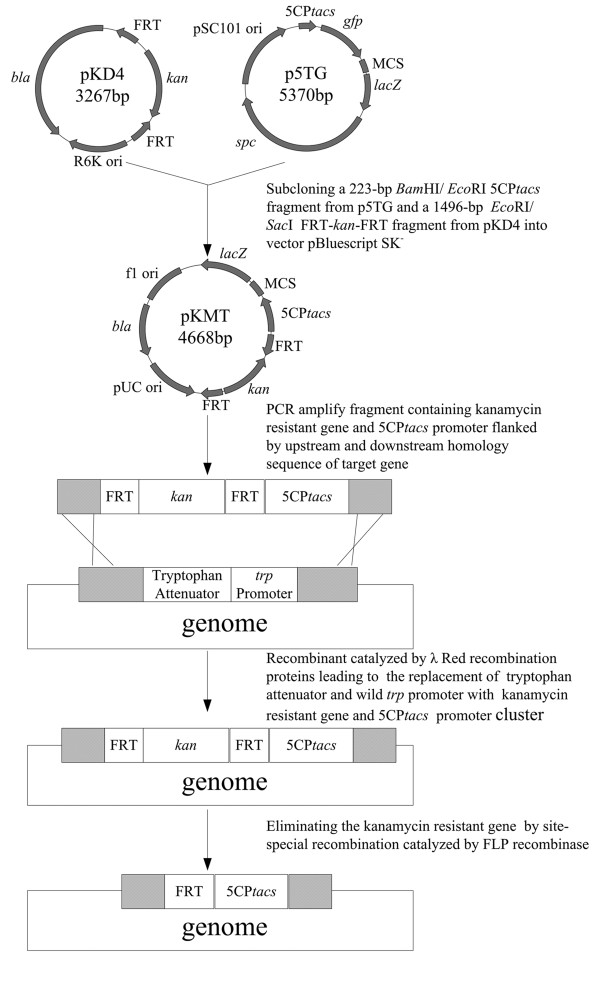
**Outline of plasmid pKMT construction and promoter swapping**.

Therefore we developed a one step attenuator inactivation and promoter swapping method (Figure 2). First, we constructed a recombinant plasmid pKMT, which contains the *kan *gene from pKD4 and 5CP*tacs *promoter cluster from p5TG. Previous work of our laboratory verified the transcription strength can be enhanced by increasing the tandem repeats of the core*-tac-*promoter and reached almost the maximum if the tandem repetitive number was five [22]. At the both side of *kan *gene, FRT sites were added. Then using this plasmid as template, the integration cassette was constructed employing PCR by adding at upstream of 5CP*tacs *promoter cluster and downstream of *kan *gene the 39 bp homologous sequences for Red recombination. Finally, by electroporating the fragments into cells of base strain *E. coli *GPT100, the engineered *E. coli *GPT101 that contains the inactivated attenuator and swapped promoter was obtained. Among twenty-four recombinants that were detected, only one positive clone was found. The positive clone was transformed with plasmid pTAT, resulting strain *E. coli *GPT1002.

### Characterization of tryptophan operon transcription in *E. Coli *GPT1002

**Figure 3:**
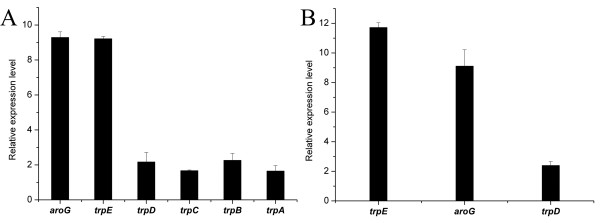
**RT-PCR analysis of constructed *E. coli***. **(A) **Relative gene expression of *E. coli *GPT1002 to the control GPT1001. **(B) **Relative gene expression of *E. coli *GPT101 to the control GPT100. *gapA *transcripts was selected as standard and each measurements were repeated three times. The error bars indicate standard deviations.

To investigate the effect of the tryptophan attenuator inactivation and promoter replacement, the transcription of five tryptophan operon genes in the strains GPT1002 and GPT1001 was compared employing RT-PCR (Figure 3A). Compared to that of the control strain GPT1001, the transcription of five tryptophan operon genes in GPT1002 was up-regulated from 1.67 ± 0.04 to 9.21 ± 0.13 times. Among that, the first gene *trpE*, direct downstream of the 5CP*tacs *promoter cluster was significantly up-regulated by 9.21 ± 0.13 times. Nevertheless, other four genes in the tryptophan operon were only about two times up-regulated. A recent publication also reported the differential expression of the genes in the same operon [23]. They found that the gene expression in the operon has linear relationship with the transcription distance. They even created a general model of operon translation to elucidate this phenomenon. However, in our study, we found that *trpD*, *trpC*, *trpB*, and *trpA *genes with different transcription distance in the operon had similar transcription level. The differences between our and their experiment is that we use the natural operon. Natural operon has some specific regulatory mechanisms, while synthetic operons used in their experiments lacked those mRNA-specific, regulatory mechanisms commonly found in native operons [23]. In native tryptophan operon, besides *trp *promoter we had swapped by 5CP*tacs *promoter cluster, there was an internal low efficiency promoter *trp p2 *located within *trpD *gene providing a bypass function advantageous to the cell under conditions of severe nutritional deprivation [24-27]. However, the regulation mechanism of promoter *trp p2 *is still unknown, which may influence the transcription of tryptophan operon and lead our novel results.

In addition, the transcription level of the *aroG *gene in the strain GPT1002 was also significantly upregulated by 9.29 ± 0.32 fold. In order to determine whether the different expression levels of *trpE *and *aroG *on the plasmid pTAT lead to this phenomenon, we analyzed the expression of *trpE*, *aroG *and *trpD *in the strain GPT101 and GPT100, the parent strains of GPT1002 and GPT1001 without the recombinant plasmid pTAT, respectively (Figure 3B). The relative transcription of three genes in both GPT101 and GPT100 were similar to the strain harboring pTAT, and therefore excluded the impact of plasmid pTAT. Since AroG protein is critical of controlling the carbon flow into aromatic amino acid biosynthesis pathway [28,29], more experiments such as metabolic flux analysis should be helpful to find out the reason of high *aroG *transcription.

### Production of L-tryptophan by *E. Coli *GPT1002

**Figure 4:**
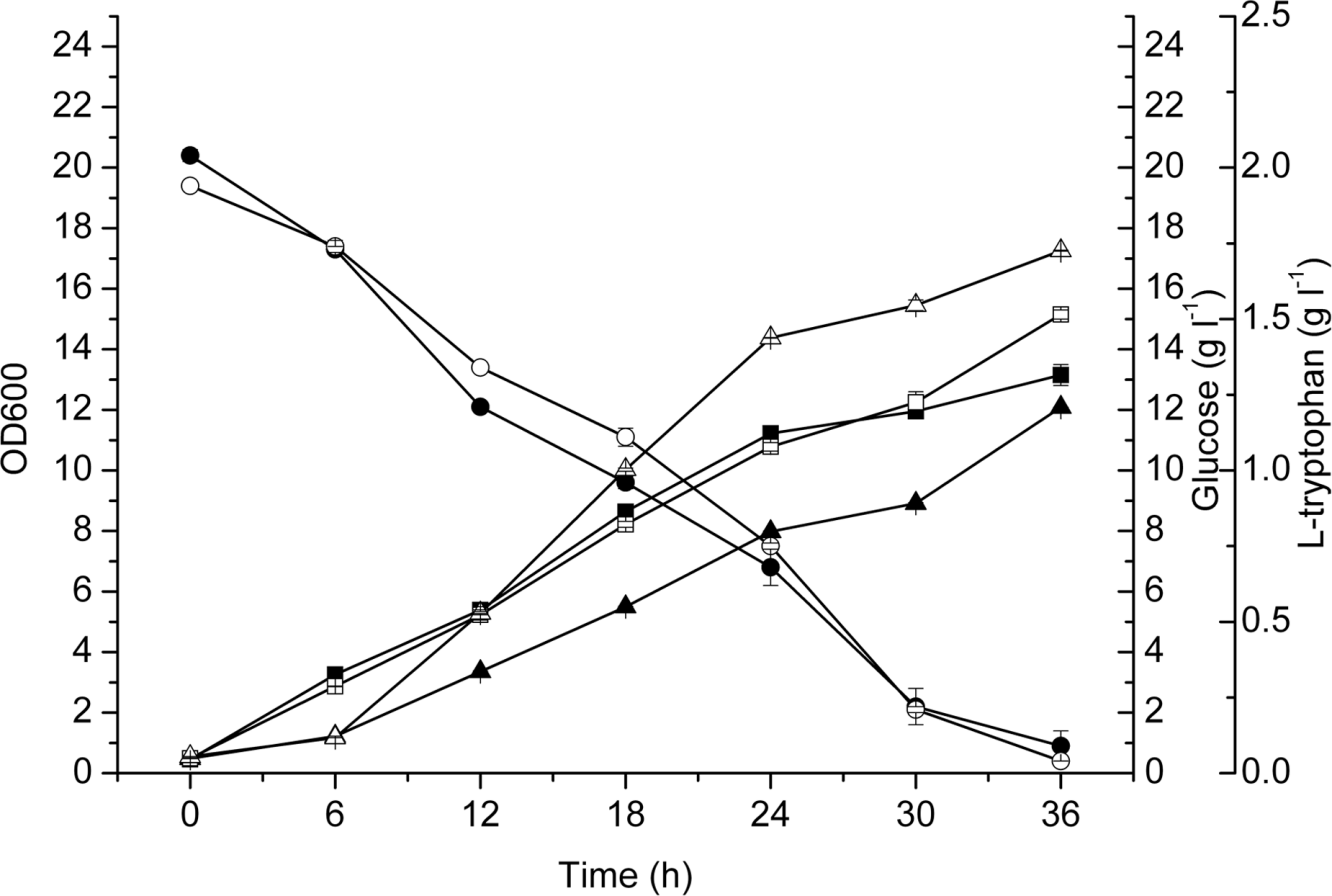
**Batch cultivation of *E. coli *GPT1001 and GPT1002 in 300-mL shake flasks**. For *E. coli *GPT1001, (filled square) growth curves; (filled circle) glucose consumption; (filled triangle) L-tryptophan yield. For *E. coli *GPT1002, (open square) growth curves; (open circle) glucose consumption; (open triangle) L-tryptophan yield. The error bars represent standard deviations from three replicate fermentations.

To explore the effect of attenuator inactivation and promoter swapping on L-tryptophan production, we performed batch cultivation of the engineered strain GPT1002 and the control GPT1001 in the medium supplemented with 20 g l^-1 ^glucose (Figure 4). Strain GPT1001 and GPT1002 showed a similar glucose consumption rate, but GPT1002 grew a little faster than the control, indicated by the optical density 600 nm (OD600) at 36 h, 15.2 vs 13.2. This implied that the genetic modification of the L-tryptophan operon may improved the glucose utilization efficiency. After 36 h cultivation, GPT1002 accumulated 1.70 g l^-1 ^L-tryptophan, 30.8% higher than that of the control strain GPT1001.

**Figure 5:**
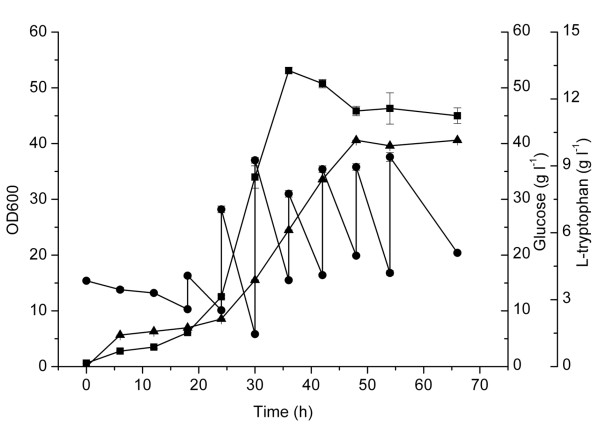
**Fed-batch fermentation of GPT1002 in 5-L fermentator**. (Filled square) growth curves; (filled circle) glucose consumption; (filled triangle) L-tryptophan yield. The error bars represent standard deviations from three measurements.

To evaluate the L-tryptophan production potential of *E. coli *strain GPT1002 under controlled conditions, we performed bioreactor fermentations under indicated cultivation condition (Figure 5). Strain GPT1002 showed a long lag growth phase of about 20 h. During this period, the L-tryptophan also accumulated at low level. This long lag growth phase may be due to the metabolic burden generated by 5CP*tacs *promoter cluster swapping and plasmid pTAT. After 20 h, the cell growth entered the exponential phase. Simultaneously, the production of L-tryptophan began to increase rapidly. The maximum OD600 was 53, while, the maximum L-tryptophan accumulation reached 10.15 g l^-1 ^at 48 h. Since GPT1002 is a genetically well-defined strain, of which the development is directly related to L-tryptophan biosynthesis, it can be easily improved by means of omics method or adapted evolution, as a lot of large-scale analytical techniques such as transcriptome and proteome analysis in this regard can be help [12,30].

Through promoter swapping, a wild type promoter could be replaced with the one that has been designed for increased or controlled transcription strength [31,32]. However, a normal strong promoter is sometimes not sufficient for downstream gene expression. Recently, we developed a promoter cluster, by which the core *tac *promoter region was arranged repetitively in tandem. This method can improve the expression of desired genes without increasing the copy number of the gene. When the repetition number was 5, the transcription strength increased almost 4 fold [22]. In this study, the 5CP*tacs *promoter cluster was swapped into the upstream region of tryptophan operon, which resulted in an ideal result. Besides *E.coli*, this promoter swapping method can also be applied in many other bacteria and other regions of the chromosome. Nevertheless, the choice of swapping region in the chromosome is very important. When the swapping region contains indispensable gene or essential regulation elements, the swapping should be careful.

## Conclusions

We developed a method for one step inactivating the tryptophan attenuator and promoter swapping. The engineered *E. coli *GPT1002 showed strong transcription capability and L-tryptophan accumulation. The L-tryptophan production of GPT1002 can be further improved through strain improvement and fermentation process optimization. The one step gene inactivating and promoter swapping is an efficient method for metabolic engineering and can also be applied in other bacteria.

## Methods

### Bacterial strains and plasmids construction

All strains, plasmids and oligonucleotides used in this study were listed in Table 2 and Table 3. *E. coli *K-12 W3110 was selected for engineering of the basic L-tryptophan-synthetic strain. *E.coli *strain DH5α was used as the host of recombinant DNA manipulation. Plasmid pCL1920 [33] was used to construct pTAT. Site-directed mutation of *trpE *(Met293Thr) and *aroG *(Pro150Leu) encoding component I of anthranilate synthase and 3-deoxy-D-arabino-heptulosonate-7-phosphate synthase respectively were performed by Easy Mutagenesis System from TransGene Biotech (Beijing, China) according to the manufacturer. Successful mutations were verified by sequencing in the BioSune Company (Shanghai, China). The *trpE^FR^*, *aroG^FR^*, and *tktA *fragments were amplified by PCR using trpE^FR^-F and trpE^FR^-R, aroG^FR^-F and aroG^FR^-R, and tktA-F and tktA-R as the primers respectively. Then the three genes were digested with *Hin*dIII/*Pst*I, *Pst*I/*Bam*HI, and *Bam*HI/*Sac*I (Fermentas) separately and ligated into cloning vector pCL1920 by T4 ligase (New England Biolabs, USA) in turn, obtaining the recombinant plasmid pTAT.

**Table 2:** Strains and plasmids used in this study

Strains	Genotype	Reference
W3110	*F^-^*, *λ^-^*, *rph-1*, *IN *(*rrnD*, *rrnE*)	Lab stock
DH5α	*F^-^*, *endA1*, *hsdR17 *(*r_K_^-^*, *m_K_^+^*), *supE44*, *thi-l*, *λ^-^*, *recA1*, *gyrA96*, Δ*lacU169 *(*Φ80lacZ *Δ*M15*)	Lab stock
GPT100	W3110 Δ*trpR*::FRT, Δ*tnaA*::FRT, Δ*ptsG*::FRT	This study
GPT101	GPT100 with tryptophan attenuator deletion and *trp *promoter swapping by 5CP*tacs *promoter cluster	This study
GPT1001	GPT100 containing pTAT	This study
GPT1002	GPT101 containing pTAT	This study
Plasmids	Genotype	Reference

pBluescript SK^-^	Ap^R^	Lab stock
pCL1920	Spc^R^	[33]
pTAT	pCL1920 containing *aroG^FR^, trpE^FR^*, and *tktA*	This study
pKD4	*bla*, FRT-*cat*-FRT	[34]
pKD3	*bla*, FRT-*kan*-FRT	[34]
pKD46	*bla*, helper plasmid	[34]
pCP20	*bla *and *cat*, helper plasmid	[35]
p5TG	pCL1920 containing 5CP*tacs *promoter cluster and *gfp*	[22]
pKMT	pBluescript SK^-^, containing *kan *and 5CP*tacs *promoter cluster	This study

**Table 3:** Primers used in this study

Primers	Nucleotide sequence
trpR-F	5'-GGACGTCGTTACTGATCCGCACGTTTATGATATGCTATCGTGTAGGCTGGAGCTGCTTC-3'
trpR-R	5'-ACGGGTATTTGTAGGACGGATAAGGCGTTCACGCCGCATATGGGAATTAGCCATGGTCC-3'
tnaA-F	5'-CAGATCAACGGCTGTACCGTGCGTAACGTCTATATCAAAGTGTAGGCTGGAGCTGCTTC-3'
tnaA-R	5'-TGTTTACCGGTTTTCGGATCGCGGCCTAACAGGAAAGAGATGGGAATTAGCCATGGTCC-3'
ptsG-F	5'-ACGTAAAAAAAGCACCCATACTCAGGAGCACTCTCAATTGTGTAGGCTGGAGCTGCTTC-3'
ptsG-R	5'-AGCCATCTGGCTGCCTTAGTCTCCCCAACGTCTTACGGAATGGGAATTAGCCATGGTCC-3'
kan-F	5'-ATAGGATCCGTGTAGGCTGGAGCTGCTTC-3'
kan-R	5'-GGGGAATTCATGGGAATTAGCCATGGTCC-3'
Mtac-F	5'-GGGGAATTCTTGACAATTAATCATCGGCTCGTATA-3'
Mtac-R	5'-GGGGTCGACATGCATCTAGTATTTCTCCTCTTTAATGGAT-3'
trp-F	5'-GTGCAGGTCGTAAATCACTGCATAATTCGTGTCGCTCAAGTGTAGGCTGGAGCTGCTTC-3'
trp-R	5'-GCAGGTTAGCAGTTCGAGAGTCGGTTTTTGTGTTTGCATATGCATCTAGTATTTCTCCT-3'
trp test-F	5'-GCCTTACCGCCAGAATGATGAATGA-3'
trp test-R	5'-GCAGCACACGGCAGTTTGGTGATTG-3'
trpRtest-F	5'-GTGCTGGCTTATGACGCTTACTACCGCTAT-3'
trpRtest-R	5'-CGCTGAGTCCGTTTCATAATGCCGTGTA-3'
tnaAtest-F	5'-TATCAATACACCATTCCGACTCACC-3'
tnaAtest-R	5'-GTGAAGTGACGCAATACTTTCGGTT-3'
ptsGtest-F	5'-CCTGTACACGGCGAGGCTCT-3'
ptsGtest-R	5'-AATAACACCTGTAAAAAAGGCAGCC-3'
trpE^FR ^-F	5'-GCCAAGCTTAAGGAGATATAATGCAAACACAAAAACCGAC-3'
trpE^FR ^-R	5'-ATTAACTGCAGTCAGAAAGTCTCCTGTGCATGATGCG-3'
aroG^FR ^-F	5'-GGGGCCTGCAGAAGGAGATATAATGAATTATCAGAACGACG-3'
aroG^FR ^-R	5'-TTATTGGATCCTTACCCGCGACGCGCTTTTACTGCATT-3'
tktA-F	5'-GGCGGATCCAAGGAGATATAATGTCCTCACGTAAAGAG-3'
tktA-R	5'-CGTGAGCTCTTACAGCAGTTCTTTTGCTTTCGCAACAACG-3'
Primers for RT-PCR	
trpERT-F	5'-CACAATCCAGGCACTTTCCG-3'
trpERT-R	5'-GGCGTCTTCATCCAGCAGTG-3'
trpDRT-F	5'-GTGCTGATGCTTTCTCCTGG-3'
trpDRT-R	5'-CCTGATGTCCGAGGCAAATG-3'
trpCRT-F	5'-CAGACAAGGCGATTTGGGTA-3'
trpCRT-R	5'-TTGACGGCGACGCTTTCTTG-3'
trpBRT-F	5'-GGCAGGCGTTGCTGGCGAAG-3'
trpBRT-R	5'-GTTAGGCGACTGGCGTTCAA-3'
trpART-F	5'-TCTGTTTGCCCAGTTGAAGG-3'
trpART-R	5'-GGGATACCTAACTCCAGCG-3'
aroGRT-F	5'-TGGGCTGGAAAGGGCTGATT-3'
aroGRT-R	5'-GAGAAACTCACCTGCCGCTG-3'
gapART-F	5'-AACTGAATGGCAAACTGACTGGTA-3'
gapART-R	5'-TTTCATTTCGCCTTCAGCAGC-3'

Plasmid pBluescript SK^- ^was served for constructing recombinant vector pKMT. The *kan *gene and the 5CP*tacs *promoter cluster were obtained with the kan-F and kan-R, and Mtac-F and Mtac-R as the primers and the plasmids pKD4 and p5TG as the templates separately using the TransTaq DNA Polymerase High Fidelity from TransGene Biotech (Beijing, China). Next, the PCR products were digested with *Bam*HI/*Eco*RI and *Eco*RI/*Sac*I respectively, and then ligated into the vector pBluscript SK^- ^and constructed the plasmid pKMT.

### Gene inactivation

Three genes *trpR*, *tnaA*, and *ptsG*, which encoded trp operon repressor, tryptophanase, and glucose-specific PTS enzyme IIBC components respectively, were inactivated in turn using the one-step inactivation method [34]. Primers trpR-F and trp-R, tnaA-F and tnaA-R, and ptsG-F and ptsG-R, template plasmids pKD3 for *ptsG *and pKD4 for *trpR *and *tnaA *were used to obtain the linearized DNA flanked by FLP recognition target sites and homologous sequences for genes deletion. The PCR was performed in an automated thermocycler (Bio-Rad, Hercules, CA, USA), and then PCR products were gel-purified and digested with *Dpn*I. Electroporation was done according to the manufacturer's instructions by using 25 ml of cells and 10-100 ng of PCR product to transform resistance gene cassette into the cells expressing the Red recombinase before. Shocked cells were added to 1 ml SOC cultures, incubated 1 h at 37°C, and one-half was spread onto agar to select chlorampenicol resistant or kanamycin resistant transformants. Positive clones on the plates were verified by PCR using the primers trpRtest-F and trpRtest-R, tnaAtest-F and tnaAtest-R, and ptsGtest-F and ptsGtest-R separately. The chlorampenicol or kanamycin cassette was removed with the helper plasmid pCP20. The final strain *E. coli *K-12 W3110 with three mutations (Δ*trpR *Δ*tnaA *Δ*ptsG*) was named GPT100.

### One-step of L-tryptophan attenuator inactivation and promoter swapping

The DNA fragment for next promoter replacement was amplified using plasmid pKMT as the template with the primers Trp-F and Trp-R and the fragment containing *kan *gene and 5CP*tacs *promoter cluster was transformed into GPT100 by electroporation, incubated for 1 hours at 37°C, and spread onto agar to select kanamycin resistant transformants. The strategy of plasmid pKMT construction and promoter swapping were listed in Figure 2. The positive clones were verified by PCR using the primers trptest-F and trptest-R, and named GPT101. Then we transformed the plasmid pTAT into GPT101 and the GPT100 respectively, and resulting to the recombinant strain GPT1002 and control strain GPT1001 for next experiments.

### Quantitative real-time reverse transcription (RT)-PCR analysis

Samples for mRNA preparation were cultivated 6 h after the addition of 0.1 mM IPTG if necessary. Total cellular RNA was extracted by the RNA simple Total RNA Kit (TIANGEN, Beijing, China) as described by the manufacturer. The quantity and purity of RNA were determined by spectrophotometrically at A_260 _and A_280_. The reverse transcription was performed using primers Random 6 mers and Oligo dT by the PrimeScript RT reagent Kit (TaKaRa, China) according to the manufacturer. RT- PCR was performed with SYBR Premix Ex TaqII (TaKaRa, China) followed the protocol of the Real-Time PCR Detection Systems (Bio-Rad, Hercules, CA, USA). The RT-PCR measurement was repeated three times for each sample. The *trpE*, *trpD*, *trpC*, *trpB*, *trpA*, *aroG *genes transcripts primers were listed in Table 3 and *gapA *encoding D-glyceraldehyde-3-phosphate dehydrogenase transcript selected as internal standard was amplified with gapART-F and gapART-R.

### Growth conditions

Strains for cloning and inoculums were grown in Luria-Bertani media (1% tryptone, 0.5% yeast extract and 1% NaCl) at 37°C for 8-12 h supplemented with the appropriate antibiotic (ampicillin (100 mg l^-1^), chloramphenicol (17 mg l^-1^), kanamycin (25 mg l^-1^), spectinomycin (50 mg l^-1^)) when necessary. For fermentation, the seed medium contained (per liter) glucose (20 g), MgSO_4_·7H_2_O (5 g), KH_2_PO_4 _(1.5 g), (NH_4_)_2_SO_4 _(10 g), yeast extract (15 g), FeSO_4_·7H_2_O (15 mg), sodium citrate dehydrate (0.5 g), Vitamin B_1 _(100 mg). The fermentative medium contained (per liter) glucose (20 g), MgSO_4_·7H_2_O (5 g), KH_2_PO_4 _(2 g), (NH_4_)_2_SO_4 _(4 g), yeast extract (1 g), FeSO_4_·7H_2_O (100 mg), sodium citrate dehydrate (2 g). A single clone was pre-cultured in 5 ml Luria-Bertani medium at 37°C and on a rotary shaker at 200 rpm overnight. 1 ml overnight cells were inoculated into 50 ml seed medium and cultured for 8-12 hours, and then 10% (v/v) seed cultures for batch cultivation were incubated into 50 mL fermentation medium at 37°C with the initial glucose concentration 20 g l^-1^. Isopropyl β-D-1-thiogalactopyranoside (IPTG) was added at the final concentration of 0.2 mM. For fed-batch fermentation, a stirred 5-l glass vessel with the BioFlo310 modular fermentor system (New Brunswick Scientific, Edison, NJ, USA) was used. The inoculum ratio was 10% (v/v). When glucose concentration in the medium was below 10 g l^-1^, feeding solution containing 500 g l^-1 ^glucose was supplied to the medium. The culture temperature was 37°C, and the pH was controlled at 6.8 with NH_3_·H_2_O. The dissolved oxygen concentration was kept at 30% via changing fermentor agitation speed and aeration rate.

### Analytical methods

Cell growth was monitored by OD600 with a spectrophotometer (Shimazu, Japan). Glucose was quantitatively analyzed by high-performance liquid chromatography (HPLC; Shimazu, Japan) equipped with a column of Aminex HPX-87H Ion Exclusion particles (300 mm × 7.8 mm, Bio-Rad, Hercules, CA, USA). Samples were centrifuged at 12 000 rpm for 5 min and then filtrated with a 0.22 μm aqueous membrane. The mobile phase was 5 mM sulfuric acid (in Milli-Q water) with the flow of 0.6 ml min^-1 ^and the column was maintained at 65°C. L-tryptophan was determined by the method of fluorometric determination [36].

## Competing interests

The authors declare that they have no competing interests.

## Authors' contributions

PG carried out most of the experiments and wrote the manuscript. PG and FY carried out the RT-PCR experiments. PG, FY and QW constructed the plasmids and strains. PG, FY and JK performed batch cultivation and bioreactor fermentation. QQ conceived of the study, participated in its design, and drafted the manuscript. All authors read and approved the final manuscript.
